# Assessing Dry Weight of Hemodialysis Patients via Sparse Laplacian Regularized RVFL Neural Network with L_2,1_-Norm

**DOI:** 10.1155/2021/6627650

**Published:** 2021-02-04

**Authors:** Xiaoyi Guo, Wei Zhou, Qun Lu, Aiyan Du, Yinghua Cai, Yijie Ding

**Affiliations:** ^1^Hemodialysis Center, The Affiliated Wuxi People's Hospital of Nanjing Medical University, 214000 Wuxi, China; ^2^Nursing Department, The Affiliated Wuxi People's Hospital of Nanjing Medical University, 214000 Wuxi, China; ^3^School of Electronic and Information Engineering, Suzhou University of Science and Technology, 215009 Suzhou, China

## Abstract

Dry weight is the normal weight of hemodialysis patients after hemodialysis. If the amount of water in diabetes is too much (during hemodialysis), the patient will experience hypotension and shock symptoms. Therefore, the correct assessment of the patient's dry weight is clinically important. These methods all rely on professional instruments and technicians, which are time-consuming and labor-intensive. To avoid this limitation, we hope to use machine learning methods on patients. This study collected demographic and anthropometric data of 476 hemodialysis patients, including age, gender, blood pressure (BP), body mass index (BMI), years of dialysis (YD), and heart rate (HR). We propose a Sparse Laplacian regularized Random Vector Functional Link (SLapRVFL) neural network model on the basis of predecessors. When we evaluate the prediction performance of the model, we fully compare SLapRVFL with the Body Composition Monitor (BCM) instrument and other models. The Root Mean Square Error (RMSE) of SLapRVFL is 1.3136, which is better than other methods. The SLapRVFL neural network model could be a viable alternative of dry weight assessment.

## 1. Introduction

Fluid overload in patients with chronic renal failure is closely related to poor cardiovascular outcomes [1, 2]. Maintenance of hemodialysis (HD) is the main method for patients with renal failure [3]. However, the accurate assessment of body water volume is still a concern [4]. At present, dry weight has been used as an important indicator to assess the homeostasis of fluids in hemodialysis patients. Medical staff can use the patient's dry weight to estimate the amount of water needed for dialysis during hemodialysis. The conventional clinical-based dry weight assessment method is time-consuming and labor-intensive [1]. There are already some methods based on bioelectrical impedance analysis (BIA) [5] to determine dry weight, including body composition monitor (BCM) [6] and lung ultrasound (LUS). However, all the above methods require special instruments and professional technicians to complete. Medical staff can use some clinical data to build predictive models [7] to accurately assess dry weight. Currently, machine learning (ML) or deep learning has solved many common clinical problems in medicine, such as brain diseases [8–10], cancer analysis, and diabetes.

Some scholars have used artificial neural networks (ANN) to predict the total water volume of hemodialysis patients and have obtained better results than conventional clinical calculation equations [11]. In addition, deep learning methods are also emerging in clinical diagnosis, including pixel-based convolutional neural networks to diagnose skin cancer [12]. In the biological field, microbiology analysis [13], CircRNAs [14], microRNAs, and cancer association prediction [15–17], lncRNA-miRNA association prediction, O-GlcNAcylation site prediction [18], DNA methylation site [19–21], protein remote homology [22], function prediction of proteins [23–29], electron transport proteins [30], breast cancer [31], cell-specific replication [32], osteoporosis diagnoses [33], and drug complex network analysis [34–38].

In our previous research, a Multiple Kernel Support Vector Regression (MKSVR) [39] predictor was proposed to assess the dry weight and obtain good predictive performance. Inspired by the previous work and baseline Random Vector Functional Link (RVFL) network [40], we propose a new dry weight assessment model, called Sparse Laplacian regularized RVFL neural network with L_2,1_-norm (SLapRVFL), which considers the topological relationship between samples and more sparse connections between the input layer and the hidden layer.

## 2. Materials and Methods

### 2.1. Materials

This work collects demographic and anthropometric data and bioimpedance spectroscopy (BIS) from historical data (2018-9 to 2019-9) from Wuxi people's hospital and the northern Jiangsu people's hospital. This study has been approved by the ethics committees of the hospitals (Nos. KYLLKS201813 and 2018KY-001). The collected patient data meet the following requirements: age greater than 18 years; ESRD for more than three months and maintenance hemodialysis [41]; no heart failure, no metal implants, no pregnancy, no disability, no infection, and no edema and other diseases; and hemodialysis treatment 3 times a week, 4 hours each time. Finally, we obtain a data set of 476 hemodialysis patients. DW is the normal body weight after clinical diabetes. DW is obtained by a clinician under strict clinical supervision using a clinical scoring system (using trial and error method) [42, 43].

We choose 7 features, including age, gender (binary feature), systolic blood pressure (SBP), diastolic blood pressure (DBP), body mass index (BMI), heart rate (HR), and years of dialysis (YD) to build our predictive model.  Table 1 shows the information of the data set. BMI is measured before hemodialysis treatment.

### 2.2. Methods

**Figure 1:**
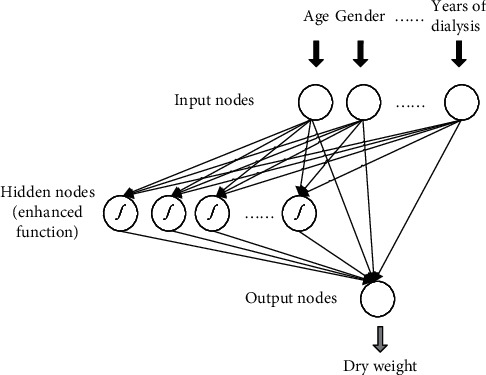
Schematic of our proposed method.

The baseline RVFL was proposed for regression or classification. The schematic diagram of RVFL is shown in Figure 1. The basic information of the patient is put into the RVFL neural network model for processing, and the predicted dry weight is the output.

Suppose, there are *N* training samples with {*x*_*i*_, *y*_*i*_}, *i* = 1, 2, ⋯, *N*. The output value is *y*_*i*_ ∈ *R*^1×*c*^ and the input data is *x*_*i*_ ∈ *R*^1×*d*^. *d* denotes the dimension of *x*_*i*_. As per Figure 1, RVFL randomly initializes all weights and deviations between the hidden layer and the input layer. These parameters are fixed during the training process and do not need to be tuned. There are connections between the output layer, input layer, and hidden layer. This part of the weight needs to be obtained by training RVFL. The output layer of RVFL is connected to both the input layer and the hidden layer, so as to ensure the nonlinear and linear relationships between the input and the output. The RVFL network with *P* hidden nodes are formulated as

(1) $$\begin{array}{c} H\beta =Y, \end{array}$$

where *β* denotes the output weight matrix; *H* is the concatenated matrix, which combines the output of the hidden layer and the input layer; and *Y* denotes the label matrix. *H* and *β* can be represented as

(2) $$\begin{array}{c} H=\left[H_{1}\;H_{2}\right], \end{array}$$

(3) $$\begin{array}{c} H_{1}={\left[\begin{array}{ccc} x_{11} & \cdots & x_{1d}\\ \vdots & \ddots & \vdots \\ x_{N1} & \cdots & x_{Nd} \end{array}\right]}_{N\times d}, \end{array}$$

(4) $$\begin{array}{c} H_{2}={\left[\begin{array}{ccc} G\left(a_{1}x_{1}+b_{1}\right) & \cdots & G\left(a_{P}x_{1}+b_{P}\right)\\ \vdots & \ddots & \vdots \\ G\left(a_{1}x_{N}+b_{1}\right) & \cdots & G\left(a_{P}x_{N}+b_{P}\right) \end{array}\right]}_{N\times P}, \end{array}$$

(5) $$\begin{array}{c} \beta ={\left[\begin{array}{c} \beta_{1}^{T}\\ \beta_{2}^{T}\\ \vdots \\ \beta_{d+P}^{T} \end{array}\right]}_{\left(d+P\right)\times C}. \end{array}$$

In Equation (4), *a*_*j*_ and *b*_*j*_ are the weights and bias of the hidden and input layers. *C* and *P* are numbers of output and hidden layer nodes. In general, the activation function is a Gaussian function: *g*(*x*) = *e*^−*x*^2^^. The activation function has a nonlinear approximation effect. To consider the potential linear relationship between the input data and the output value, RVFL adds a direct connection weight between the input layer and the output layer. Therefore, RVFL is a model that contains both linear and nonlinear approximations to improve prediction performance. For optimal *β*, the RVFL can be formulated as a regularized least-squares:

(6) $$\begin{array}{c} \beta^{*}=\arg\min\;\frac{1}{2}{\left\|H\beta -Y\right\|}_{2}^{2}+\frac{\lambda }{2}{\left\|\beta \right\|}_{2}^{2}, \end{array}$$

where *λ* is the parameter of regularization term. The solution of Equation (6) can be found by setting its gradient to 0:

(7) $$\begin{array}{c} \beta^{*}={\left(H^{T}H+\lambda I\right)}^{-1}H^{T}Y, \end{array}$$

where *I* denotes the identity matrix. However, the RVFL network did not consider the topological relationship between samples. For the output node, it must be connected to both the input and the hidden layer.

In order to further improve the robustness of RVFL, we propose Sparse Laplacian regularized RVFL neural network with L_2,1_-norm (SLapRVFL). The objective function is

(8) $$\begin{array}{c} \beta^{*}=\arg\min\;\frac{1}{2}{\left\|H\beta -Y\right\|}_{2}^{2}+\frac{\lambda_{1}}{2}Tr\left({\left(H\beta \right)}^{T}LH\beta \right)+\frac{\lambda_{2}}{2}{\left\|\beta \right\|}_{2,1}^{2}, \end{array}$$

where *L* ∈ *R*^*N*×*N*^ denotes the Laplacian matrix. *λ*_1_ and *λ*_2_ are the coefficients of Laplacian regularization the and L_21_-norm term, respectively. Laplacian regularization is used to indicate the potential manifold between samples. It can better describe the topological association between samples to improve the generalization ability of the model. Since the third term of ‖*β*‖_2,1_^2^ is not diversified, we convert Equation (8) to

(9) $$\begin{array}{c} \beta^{*}=\arg\min\;\frac{1}{2}{\left\|H\beta -Y\right\|}_{2}^{2}+\frac{\lambda_{1}}{2}Tr\left({\left(H\beta \right)}^{T}LH\beta \right)+\frac{\lambda_{2}}{2}Tr\left(\beta^{T}G\beta \right), \end{array}$$

where *G* ∈ *R*^(*d* + *P*)×(*d* + *P*)^ denotes a diagonal matrix whose *i*th-diagonal element

(10) $$\begin{array}{c} G_{ii}=\frac{1}{2{\left\|\beta_{i}\right\|}_{2}},\;i=1,2,\cdots ,\left(d+P\right). \end{array}$$

We take the derivative of the formula Equation (10) as

(11a) $$\begin{array}{c} H^{T}\left(H\beta -Y\right)+\lambda_{1}H^{T}LH\beta +\lambda_{2}G\beta =0, \end{array}$$

(11b) $$\begin{array}{c} H^{T}H\beta +\lambda_{1}H^{T}LH\beta +\lambda_{2}G\beta =H^{T}Y, \end{array}$$

(11c) $$\begin{array}{c} \left(H^{T}H+\lambda_{1}H^{T}LH+\lambda_{2}G\right)\beta =H^{T}Y, \end{array}$$

(11d) $$\begin{array}{c} \beta ={\left(H^{T}H+\lambda_{1}H^{T}LH+\lambda_{2}G\right)}^{-1}H^{T}Y. \end{array}$$

We use the baseline RVFL solution with Equation (7) as the initial *β*^0^. In addition, the Laplacian matrix can be calculate as

(12a) $$\begin{array}{c} L=D^{-1/2}\Delta D^{-1/2}, \end{array}$$

(12b) $$\begin{array}{c} \Delta =D-S, \end{array}$$

where *D* is diagonal matrix, *D*_*ii*_ = ∑_*j*=1_^*N*^*S*_*ij*_. Similarity matrix *S* is built by Radial Basis Function (RBF):

(13) $$\begin{array}{c} S_{ij}=\exp\left(-\gamma {\left\|x_{i}-x_{j}\right\|}^{2}\right). \end{array}$$

The process of SLapRVFL is list in Algorithm 1.

## 3. Results

We test our model on the benchmark data set and obtain the optimal parameters of the predictor through cross-validation. The SLapRVFL network is compared to other machine learning-based models. In addition, the body composition monitor (BCM) device (Fresenius Medical Care, Baden Humboldt, Germany) is also compared with the SLapRVFL network.

### 3.1. Evaluation Measurements

The 10-fold cross-validation (10-CV) is employed to evaluate the robustness of methods. Root Mean Square Error (RMSE), *R* square, correlation coefficient (*R*), Bland–Altman analysis, and Empirical Cumulative Distribution Plot (ECDP) [44] are all used in our study. To evaluate the agreement of two different methods, the Bland–Altman analysis usually can obtain whether the two methods can be substituted for each other (equivalence). Evaluating the agreement of the two methods can answer the question, “Can these two methods replace each other?”

### 3.2. Selection of Optimal Parameters

**Figure 2:**
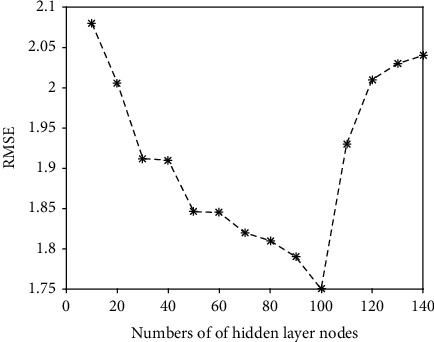
The RMSE under different numbers of hidden layer nodes (SLapRVFL network).

To get the optimal parameters of the predictive method, we obtain them through a grid search method. The parameters that need to be determined include the numbers of hidden layer nodes *P*, maximum iterations, and coefficients of *λ*_1_ and *λ*_2_. For the numbers of hidden layer nodes *P*, we fix the iterations, *λ*_1_ and *λ*_2_. Setting the maximum number as 50, *λ*_1_ = 1 and *λ*_2_ = 1. The value of *P* is from 10 to 140 with step of 10. The results are shown in Figure 2. From 10 to 100, the more neurons in the hidden layer, the lower the RMSE. Since then, RMSE has gradually increased. So, we get the lower RMSE under *P* = 100.

**Figure 3:**
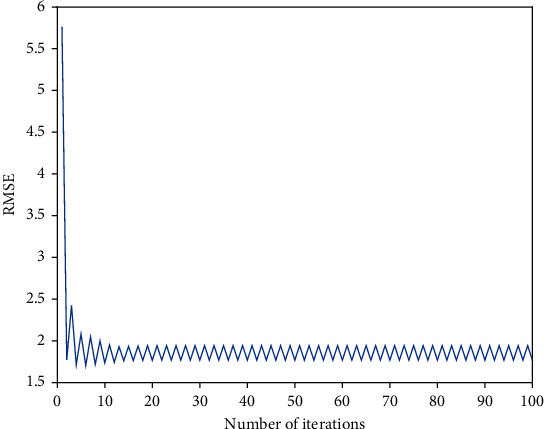
The RMSE of iterations on the training set.

Next, *P* = 100, *λ*_1_ = 1, and *λ*_2_ = 1. We gradually increase the number of iterations from 1 to 100 (shown in Figure 3). After the number of iterations reaches 10, the RMSE value drops to a minimum and slightly oscillates within a certain value. In our study, maximum number of iterations is 10.

**Figure 4:**
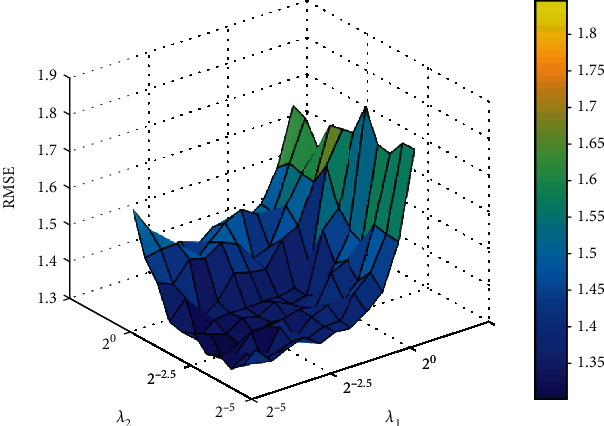
The RMSE under different *λ*_1_ and *λ*_2_.

Then, we use the better number of hidden layer nodes and iterations to search for the best *λ*_1_ and *λ*_2_. The search range of parameters is from 2^−5^ to 2^0^ (with step of 2^0.5^).  Figure 4 shows the results of different parameters. When *λ*_1_ and *λ*_2_ are 2^−3^ and 2^−2.5^, RMSE is the lowest.

### 3.3. Comparison to Other Predictive Models and BCM

**Figure 5:**
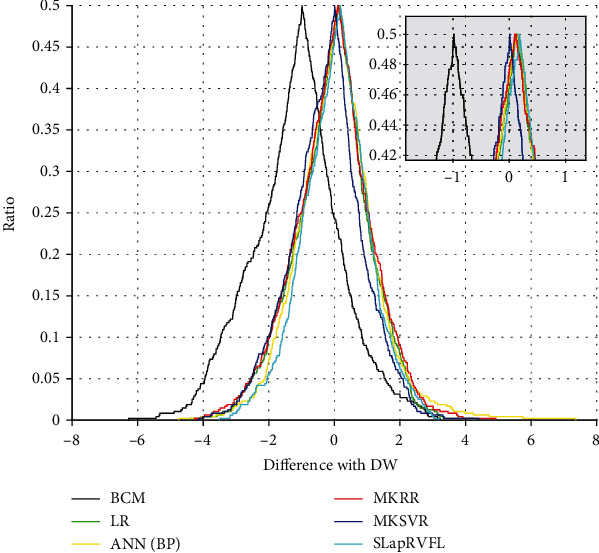
Folded empirical cumulative distribution plot between different methods.

To evaluate our model, SLapRVFL is compared with our previous work of Multiple Kernel Support Vector Regression (MKSVR) [39], Multikernel Ridge Regression (MKRR), Linear Regression (LR), Artificial Neural Network based on Back Propagation algorithm (ANN with BP), and BCM measuring instrument. Clinical dry weight is our reference standard (also the regression target value of the prediction model). The comparisons are listed in Table 2, which shows that SLapRVFL achieves best performance of RMSE (1.3136). Although the ECDP median value (peak) of MKSVR (0.0082) is more close to zero, Figure 5 shows that SLapRVFL has the least bias and much less tails than MKSVR (smaller width). The RMSE of BCM is 1.9694, which is larger than SLapRVFL.

### 3.4. Bland–Altman Analysis

**Figure 6:**
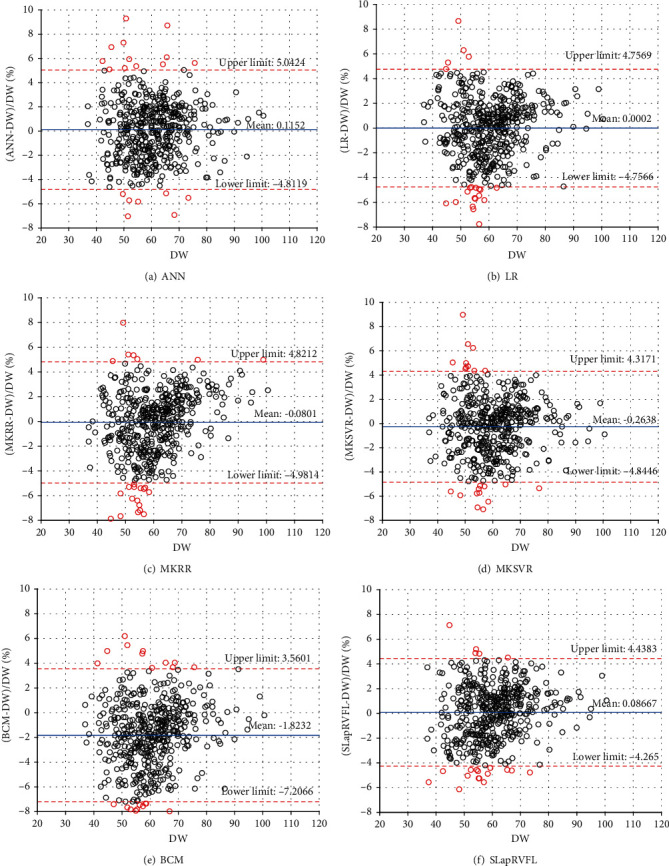
Bland–Altman plot analysis.

Bland–Altman plot is a useful tool to evaluate the agreement between predictive methods and clinical DW. In Table 3 and Figure 6, SLapRVFL, MKSVR, LR, ANN (BP), MKRR, and BCM are analyzed via Bland-Altman difference plot. SLapRVFL achieves the smallest range of 95% confidence interval (-0.1133 to 0.2866) and standard deviation (2.2202). In addition, the number (ratio) of outside agreement interval for predictive models is all less than 24 (5%) predictive samples. These results of models are clinically acceptable. SLapRVFL achieves least number (20) of the outside agreement interval in Table 3. As shown in Figure 6, two red horizontal dotted lines (upper and lower) denote the upper and lower limits of the 95% agreement limit, respectively. The middle blue solid line is the average value of the difference (between measurement methods and clinical DW). While one measurement method and clinical method can be considered as a better agreement, they can be substituted for each other (equivalence). If 95% of the points of the data set are in the agreement range, the measurement method (predictive model) is clinically acceptable. The results of the evaluation show that SLapRVFL can help clinicians assess DW with low cost.

## 4. Discussion

Due to the limitations of clinical and BCM measurement (more time and cost), this study uses a machine learning method to assess the dry weight of hemodialysis patients. Based on the basic RVFL, we propose a sparse Laplace regularized RVFL network (SLapRVFL) model. SLapRVFL is compared not only with other machine learning methods (such as LR, MKRR, ANN with BP, and MKSVR) but also with BCM equipment (commonly used in hospitals). The RMSE and Bland–Altman analysis of the model are better than the BCM instrument. It is proven that the predictive model driven by data can provide reference for clinical dry weight assessment.

BCM requires the patient's information on weight (before hemodialysis) and height. It is a portable, inexpensive, and noninvasive technology that has been used to measure DW [45, 46]. For the Bland–Altman analysis, SLapRVFL achieves the least number (20) of outside agreement interval. However, BCM has 30/476 (6.30%) points (ratio) of the outside agreement interval. Obviously, our method has better agreement with the clinical method.

## 5. Conclusions

To further improve the robustness of RVFL, we introduce sparse Laplacian regular term with L_2,1_-norm. In the training process, the graph topology information and the sparse weight matrix (output) are employed to improve the robustness of the RVFL. In fact, our work provides a new idea for assessing patients' dry weight. Not only that, in the fields of biology [47–57], pharmacy [58], and medicine [12, 59, 60], machine learning methods have helped solve many analysis tasks. In future research, we will consider collecting more samples, introducing more patient personal information, and building a predictor based on a deep learning model to more accurately assess the dry weight of hemodialysis patients.

## Figures and Tables

**Algorithm 1:**
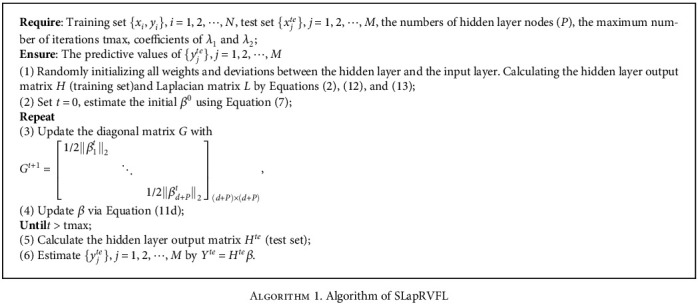
Algorithm 1. Algorithm of SLapRVFL

**Table 1:** The information of data set.

Feature	Value	*r* ^∗^
Age (years)	54.17 ± 14.22	-0.2341
Gender (males/females)	312/164	-0.4489
BMI	22.96 ± 2.95	0.9558
Systolic blood pressure (mmHg)	150.64 ± 29.36	-0.1739
Diastolic blood pressure (mmHg)	88.32 ± 19.56	-0.1249
Heart rate (times/min)	73.41 ± 8.92	0.1862
Years of dialysis (years)	5.97 ± 3.22	-0.1069

^∗^Denotes that each feature correlated with dry weight using Pearson correlation coefficient (*r*).

**Table 2:** Comparison on existing methods via 10-fold cross-validation.

Method	*R*	*R* squared	RMSE	Empirical cumulative distribution plot		
				Highest value	Lowest value	Median value
BCM^∗^	0.9473	0.9137	1.9694	3.2235	-6.2776	-0.9863
LR^∗^	0.9403	0.9308	1.4335	4.2524	-4.4014	0.1418
ANN (BP)^∗^	0.9398	0.9295	1.4794	7.3661	-4.7447	0.1324
MKRR^∗^	0.9399	0.9289	1.5015	4.9227	-4.2604	0.1104
MKSVR^∗^	0.9412	0.9321	1.3817	4.3962	-4.1273	0.0082
RVFL	0.9389	0.9300	1.3828	6.7004	-4.3557	0.0704
SLapRVFL (our method)	0.9632	0.9501	1.3136	3.1940	-3.5066	0.1014

^∗^The results are from previous work on MKSVR [39].

**Table 3:** Bland–Altman plot analysis for different models.

Model	Differences with DW (%)			Limits of agreement (%)		
	Mean	SD	95% confidence interval	Lower limit	Upper limit	Number (ratio) of outside agreement interval
BCM^∗^	-1.8232	2.7466	-2.0706 to -1.5759	-7.2066	3.5601	30/476 (6.30%)
LR^∗^	0.0002	2.4269	-0.2184 to 0.2187	-4.7566	4.7569	21/476 (4.41%)
ANN (BP)^∗^	0.1152	2.5139	-0.1112 to 0.3416	-4.8119	5.0424	22/476 (4.62%)
MKRR^∗^	-0.0801	2.5007	-0.3053 to 0.1451	-4.9814	4.8212	23/476 (4.83%)
MKSVR^∗^	-0.2638	2.3372	-0.4743 to -0.05329	-4.8446	4.3171	22/476 (4.62%)
SLapRVFL (our method)	0.0867	2.2202	-0.1133 to 0.2866	-4.2650	4.4383	20/476 (4.20%)

^∗^The results are from previous work on MKSVR [39].

## Data Availability

The data used to support the findings of this study are available from the corresponding author upon request.
